# Effects of Group 1 versus Group 2 Carbapenems on the Susceptibility of *Acinetobacter baumannii* to Carbapenems: A Before and After Intervention Study of Carbapenem-Use Stewardship

**DOI:** 10.1371/journal.pone.0099101

**Published:** 2014-06-09

**Authors:** Young Kyung Yoon, Kyung Sook Yang, Seung Eun Lee, Hyun Jeong Kim, Jang Wook Sohn, Min Ja Kim

**Affiliations:** 1 Division of Infectious Diseases, Department of Internal Medicine, Korea University College of Medicine, Seoul, Republic of Korea; 2 Institute of Emerging Infectious Diseases, Korea University College of Medicine, Seoul, Republic of Korea; 3 Department of Biostatistics, Korea University College of Medicine, Seoul, Republic of Korea; 4 Infection Control Unit, Korea University Anam Hospital, Seoul, Republic of Korea; University of Birmingham, United Kingdom

## Abstract

**Objective:**

Antimicrobial stewardship programs have been proposed for reducing bacterial resistance in the hospital environment. The purpose of this study was to investigate the impact of a carbapenem-use stewardship program on the susceptibility of *Acinetobacter baumannii* to Group 2 carbapenems.

**Methods:**

A before and after intervention study was conducted at a university hospital from September 2008 to February 2013. Three study periods were defined: Phase I, pre-intervention (months 1–18); Phase II, a postintervention period during which ertapenem use was mandated but carbapenem use was not restricted (months 19–36); and Phase III, a postintervention period during which Group 2 carbapenem use was restricted (months 37–54).

**Results:**

During the study period, intervention resulted in diminished consumption of Group 2 carbapenems (antimicrobial use density (AUD): 21.3±6.0 in Phase I, 18.8±6.0 in Phase II, 16.1±4.4 in Phase III; *P* = 0.028) and increased consumption of ertapenem (AUD: 2.7±1.7 in Phase I, 7.2±4.5 in Phase II, 9.1±5.3 in Phase III; *P*<0.001). The use of autoregressive-error models showed that in contrast with ertapenem use, the use of Group 2 carbapenem during the previous one month was positively and significantly associated with a subsequent increase in the proportion of carbapenem-resistant *A. baumannii* (CRAB) (*P* = 0.031).

**Conclusions:**

Implementing a carbapenem-use stewardship program featuring the preferential use of ertapenem for treating appropriate indications of infection resulted in reduced use of Group 2 carbapenems and had a positive impact on the susceptibility of *A. baumannii* to carbapenems. This approach could be integrated into CRAB-control strategies in hospitals.

## Introduction

Carbapenem-resistant *Acinetobacter baumannii* (CRAB) has emerged as a key nosocomial pathogen that might contribute to the spread of infection or might frequently cause outbreaks of infection [1], [2]. In recent years, carbapenem-resistant isolates have become a major concern: few therapeutic options are available for such isolates, and their infections are associated with devastating outcomes in terms of mortality, morbidity, and cost [3], [4]. Carbapenems are still one of the most widely used therapeutic options for *A. baumannii*, as long as the isolates remain susceptible to these drugs [5]. However, the incidence CRAB infection is increasing in several countries [1], [2], [6], and a major factor that has been suggested to contribute to this growing crisis is the increased use of carbapenems to treat infections caused by extended-spectrum β-lactamase (ESBL)-producing or Amp-C β-lactamase-producing gram-negative bacteria [7]–[10].

Ertapenem, the only representative of Group 1 carbapenems, exhibits limited activity against *Pseudomonas aeruginosa* and *A. baumannii*, and it is thus unlike Group 2 carbapenems (e.g., imipenem and meropenem) that are active against these pathogens. However, the growing requirement for the use of ertapenem against ESBL-producing *Enterobacteriaceae* has raised questions about the capacity of ertapenem to select for carbapenem-resistant nonfermentative gram-negative bacilli or to compromise their susceptibilities to Group 2 carbapenems [11], [12]. Conversely, both in vitro and clinical studies have supported the premise that ertapenem is less likely to select for resistance because it exhibits minimal activity against *P. aeruginosa* [13]–[18]. These studies on resistance profiles and antibiotic-prescribing practices have focused mainly on *P. aeruginosa* isolates, and limited data are currently available in the case of *A. baumannii*.

The purpose of this study was to evaluate the impact of a program of carbapenem-use stewardship on the susceptibility of *A. baumannii* to Group 2 carbapenems. In this program, the use of ertapenem instead of Group 2 carbapenems was mandated for treating appropriate indications of infection.

## Materials and Methods

### Ethics statement

The study protocol was approved by the institutional review board of Korea University Anam Hospital, and the requirement for informed consent was waived because in this study, no deviations from routine medical practices were necessary (No. AN11163).

### Hospital setting

This before-and-after study was conducted at a 950-bed tertiary-care hospital that contains two medical and one surgical intensive-care units (ICUs) (23 beds per ICU), in Seoul, Republic of Korea, between September 2008 and February 2013. Preexisting hospital infection-control practices used for multidrug-resistant organisms (MDROs) were maintained unchanged throughout the study period. These practices included contact-isolation precautions, routine environmental cleansing, and the preparation of active surveillance cultures to check for the acquisition of rectal vancomycin-resistant enterococci and also nasal and rectal methicillin-resistant *Staphylococcus aureus* (MRSA) at ICU admission and weekly thereafter in all patients who stayed for more than 24 h in the ICUs. Furthermore, the preparation of active surveillance cultures was extended to screen for nasopharyngeal CRAB in ICU patients starting from April 2012, in the middle of Phase III of this study. Between April and December 2012, we temporarily implemented the preparation of environmental surveillance cultures and extensive environmental cleansing in only the medical ICU for the purpose of containing CRAB disseminations. Moreover, the hospital used a computerized antibiotic-prescription system; infectious-disease specialists have controlled the proper use of antibiotics through such antibiotic-restriction programs since 2002 [19].

### Study design

This was a before-and-after study conducted to assess the impact of carbapenem stewardship on the susceptibility rate of CRAB isolates to carbapenems. The hospital formulary included imipenem, meropenem, panipenem, and doripenem as Group 2 carbapenems; panipenem was replaced with doripenem in May 2011. Ertapenem, a Group 1 carbapenem, was added to the hospital formulary in January 2005. All carbapenem prescriptions required the approval of an infectious-disease specialist before use.

The 54-month study period was divided into three phases and featured the following construction of a time-series analysis:

1. Phase I (September 2008 to February 2010): the pre-intervention period; the prescription of both Group 1 and 2 carbapenems was allowed as empirical therapy in the case of severe patients or as the definite therapy for patients infected with MDROs.
2. Phase II (March 2010 to August 2011): the ertapenem-mandatory period; the replacement of Group 2 carbapenems with ertapenem was mandated in the case of infections caused by ESBL-producing *Enterobacteriaceae* in patients who were not coinfected with nonfermentative gram-negative bacilli.
3. Phase III (September 2011 to February 2013): the carbapenem-restriction period; the use of Group 2 carbapenems was allowed only for treating infections caused by ESBL-producing *Enterobacteriaceae*, *P. aeruginosa*, or *A. baumannii*, with susceptibility to Group 2 carbapenem alone, or for treating hemodynamically unstable patients who did not respond to empirical antibiotic therapy.

During Phase III, our hospital implemented a new computer program in which a pop-up screen was used to communicate the detailed conditions required for carbapenem prescription in an effort to restrict carbapenem use.

### Data collection

Susceptibility data on the targeted gram-negative bacilli were collected from the hospital electronic database of clinical microbiology. In the case of repetitive isolates of the targeted species collected from a single patient during the same in-patient stay, only the first isolate was included in the analysis. Clinical cultures were prepared according to the clinician's request and were based on the patients' symptoms. Results of surveillance cultures were not included in this analysis.

The antimicrobial-susceptibility rate was measured as the proportion of susceptible isolates of each microorganism tested. The use of antimicrobial agents was measured monthly using the antimicrobial use density (AUD), which is the number of defined daily doses per 1000 patient-days; the defined daily dose was determined based on the description provided by the World Health Organization (www.whocc.no/atcddd/). The number of patient-days, incidence of all-cause deaths, and incidence of CRAB-related deaths were also evaluated during each study period.

### Microbiological methods

Identification and antimicrobial susceptibility tests of all isolates were performed by using the VITEK 2 system (bioMérieux, Hazelwood, MO, USA). The susceptibility results were interpreted according to the criteria of the Clinical and Laboratory Standards Institute (M100-S23).

### Statistical analyses

Continuous variables were expressed as means ± standard deviations or medians (ranges), and categorical variables were expressed as percentages of a specific group. Kruskal-Wallis tests and multiple comparisons using Mann-Whitney tests performed with Bonferroni adjustment were used to assess the statistical significance of the changes in antibiotic usage and antibiotic susceptibility during the three phases of the study. To evaluate changes in the proportions of isolates of multidrug-resistant gram-negative bacilli among the three phases, we used the count data to perform logistic regression analysis.

Time-series data were fitted to linear-regression models that included autoregressive errors to determine statistically significant associations between antibiotic usage and antibiotic susceptibility throughout the study period. Durbin-Watson tests for autocorrelations were used to eliminate the possibility of false-positive results that might have been generated by autocorrelations of the monthly data series. Results were considered statistically significant when *P*-values were <0.05. All analyses were performed using IBM SPSS Statistics Version 20.0 (IBM Corporation, Armonk, NY, USA), R 2.15.2 (The R Foundation for Statistical Computing, Vienna, Austria), and SAS 9.2 (SAS Institute Inc., Cary, NC, USA).

## Results

### Changes in carbapenem use

During the 54-month study period, a total of 1795 patients received the following carbapenems: ertapenem (n = 755; 42.1%), imipenem (n = 344; 19.2%), meropenem (n = 687; 38.3%), panipenem (n = 5; 0.3%), and doripenem (n = 4; 0.2%). The monthly average consumptions of total carbapenems, Group 2 carbapenems, and ertapenem were 24.1±6.1 AUD (median, 22.9; range, 14.3–40.1), 18.7±5.8 AUD (median, 18.5; range, 9.0–35.1), and 6.3±4.8 AUD (median, 5.6; range, 0.16–23.2), respectively. The magnitude of the effect of the carbapenem-use stewardship as an intervention was estimated by means of multiple comparisons performed using Bonferroni-corrected Mann-Whitney tests. Ertapenem AUD increased significantly from Phase I (2.7±1.7 AUD) to Phase II (7.2±4.5 AUD; *P*<0.001) and Phase III (9.1±5.3 AUD; *P*<0.001). By contrast, the AUDs of total carbapenem decreased from 24.0±6.6 AUD in Phase I to 25.5±7.0 AUD in Phase II (*P* = 0.521) and 22.7±4.7 AUD in Phase III (*P* = 0.252). Group 2 carbapenem AUDs decreased from 21.3±6.0 AUD in Phase I to 18.8±6.0 AUD in Phase II (*P* = 0.293) and 16.1±4.4 AUD in Phase III (*P* = 0.006). In Kruskal-Wallis tests, the respective changes were significant during the three phases in the use of both ertapenem (*P* = 0.028) and Group 2 carbapenems (*P*<0.001), indicating that the carbapenem-use stewardship was practiced and maintained throughout Phases II and III (Table 1).

**Table 1:** Changes in antibiotic use during the study period.

Phase/antibiotics	Median AUD (range)	*P*-value*
**Group 1 carbapenem (ertapenem)**		
I	2.7 (0.2–5.8)	<0.0001
II	6.5 (2.9–22.9)^a^	
III	7.2 (3.3–23.3)^a^	
**Group 2 carbapenems**		
I	20.7 (13.7–35.1)^a^	0.028
II	18.7 (9.0–30.1)^a,b^	
III	15.5 (10.0–24.9)^b^	
**Total carbapenems**		
I	23.0 (14.3–40.1)^a,c^	0.499
II	24.6 (14.9–36.4)^a,b^	
III	21.8 (16.2–35.1)^b,c^	
**Third-generation cephalosporins**		
I	102.2 (86.7–172.1)	0.311
II	98.6 (78.4–185.3)	
III	96.7 (76.4–251.8)	
**Fluoroquinolones**		
I	57.7 (51.1–68.7)	0.102
II	59.1 (49.8–116.3)	
III	67.1 (46.4–187.8)	

AUD, antimicrobial use density; the number of defined daily doses per 1000 patient-days.

*P-values were determined using Kruskal-Wallis tests.

a,b,c Same letters indicate statistical insignificance based on multiple comparisons performed using Bonferroni-corrected Mann-Whitney tests.

When carbapenem use was categorized based on clinical cultures, the proportion of culture-directed use accounted for 58.9% (n = 1055): 54.0% (319/591) in Phase I, 57.2% (361/631) in Phase II, and 65.4% (375/573) in Phase III (*P*<0.001). The most common, clinically relevant isolates were *Escherichia coli* (38.7%), followed by *K. pneumoniae* (15.8%), *A. baumannii* (15.2%), and *P. aeruginosa* (11.1%). The types of infection included urinary-tract infections (23.9%), intra-abdominal infections (20.5%), primary bacteremia (16.1%), pneumonia (14.6%), neutropenic fever (10.7%), fevers of unknown origin (9.8%), skin and soft-tissue infections (2.5%), and other infections (2.0%). No carbapenems were used for prophylactic treatment.

### Changes in clinical outcomes

During the study period, hospitalized-population density (per 1000 patient-days) was not significantly different among the phases (Phase I, 95,243.5 (87,373.0–100,866.0); Phase II, 97,147.5 (84,664.0–102,817.0); and Phase III, 97,317.0 (87,616.0–103,413.0); *P* = 0.395). Moreover, no statistically significant differences were measured between all-cause mortality rates and CRAB-related deaths before and after the intervention. The all-cause mortality rates (per 1000 patient-days) were 5.81 (3.87–7.67) in Phase I, 6.24 (4.99–7.68) in Phase II, and 5.77 (3.90–8.15) in Phase III (*P* = 0.681); the incidences of CRAB-related deaths (per 1000 patient-days) were 0.11 (0–0.61) in Phase I, 0.20 (0–0.63) in Phase II, and 0.31 (0–0.62) in Phase III (*P* = 0.220). Overall, these data suggest that the intervention might not have influenced the clinical outcomes.

### Changes in CRAB proportions

During the study period, a total of 3355 patients were colonized or infected with *A. baumannii*, as determined based on clinical cultures. From 2053 (61.2%) of these patients, CRAB isolates were obtained. The monthly incidence of CRAB colonization or infection was 0.58±0.20 (median, 0.60; range, 0.13–0.98) per 1000 patient-days. The monthly CRAB proportion was 60.7%±12.3% (median, 63.2%; range, 24.1%–84.1%). The monthly CRAB incidence (per 1000 patient-days) did not change significantly during the three phases of the study (Phase I: median, 0.05; range, 0.35–0.83; Phase II: median, 0.62; range, 0.13–0.88; Phase III: median, 0.60; range, 0.29–0.98; *P* = 0.354).

In this study, we also separately analyzed the colonized (n = 2446) and infected (n = 909) patients from whom *A. baumannii* isolates were obtained. Among the 909 patients who were infected with *A. baumannii*, 455 patients (50.1%) had CRAB isolates. The monthly proportion of CRAB isolation was 50.1%±14.0% (median, 47.5%; range, 21.7%–76.9%). When time was considered using the autoregressive-error model, the proportion of CRAB isolates in the infected patients with *A. baumannii* isolates was determined to be increased significantly during the study period (Table 2).

**Table 2:** Changes in proportions of isolates of multidrug-resistant gram-negative bacilli during the study period.

Phase/organism	No. of isolates (%)	Comparison	OR	95% CI	*P*-value*	*P*-value**
**CRAB infection or colonization**						
I	642/1229 (52.2)	II versus I	1.52	(1.28–1.80)	<0.0001	
II	630/1009 (62.4)	III versus I	2.13	(1.79–2.52)	<0.0001	<0.0001
III	781/1117 (69.9)	II versus III	0.72	(0.60–0.86)	<0.0001	
**CRAB infection**						
I	141/335 (42.1)	II versus I	1.63	(1.19–2.25)	0.003	
II	152/280 (54.3)	III versus I	1.69	(1.23–2.32)	0.001	0.001
III	162/294 (55.1)	II versus III	0.97	(0.70–1.34)	0.844	
**CRAB colonization**						
I	501/894 (56.0)	II versus I	1.49	(1.22–1.83)	<0.0001	
II	478/729 (65.6)	III versus I	2.38	(1.94–2.93)	<0.0001	<0.0001
III	619/823 (75.2)	II versus III	0.63	(0.50–0.78)	<0.0001	
**Carbapenem-resistant ** ***Pseudomonas aeruginosa***						
I	222/1226 (18.1)	II versus I	1.00	(0.81–1.24)	0.983	
II	203/1119 (18.1)	III versus I	1.09	(0.89–1.34)	0.409	0.648
III	216/1111 (19.4)	II versus III	0.92	(0.74–1.14)	0.432	
**ESBL-producing ** ***K. pneumoniae*** **, community-acquired or healthcare-associated**						
I	156/1008 (15.48)	II versus I	1.21	(0.97–1.51)	0.096	
II	223/1230 (18.13)	III versus I	2.26	(1.84–2.78)	<0.0001	<0.0001
III	390/1333 (29.26)	II versus III	0.54	(0.44–0.65)	<0.0001	
**ESBL-producing ** ***K. pneumoniae*** **, community-acquired**						
I	59/525 (11.2)	II versus I	1.10	(0.77–1.59)	0.598	
II	74/604 (12.3)	III versus I	1.16	(0.81–1.68)	0.413	0.711
III	74/576 (12.8)	II versus III	0.95	(0.67–1.34)	0.758	
**ESBL-producing ** ***K. pneumoniae*** **, healthcare-associated**						
I	97/483 (20.1)	II versus I	1.24	(0.93–1.66)	0.140	
II	149/626 (23.8)	III versus I	2.58	(2.19–3.72)	<0.0001	<0.0001
III	316/757 (41.7)	II versus III	0.44	(0.35–0.55)	<0.0001	
**ESBL-producing ** ***E. coli*** **, community-acquired or healthcare-associated**						
I	447/2855 (15.66)	II versus I	1.35	(1.18–1.54)	<0.0001	
II	609/3044 (20.01)	III versus I	1.90	(1.67–2.16)	<0.0001	<0.0001
III	829/3180 (26.07)	II versus III	0.71	(0.63–0.80)	<0.0001	
**ESBL-producing ** ***E. coli*** **, community-acquired**						
I	244/2217 (11.0)	II versus I	1.44	(1.20–1.71)	<0.0001	
II	343/2274 (15.1)	III versus I	1.92	(1.62–2.27)	<0.0001	<0.0001
III	436/2274 (19.2)	II versus III	0.75	(0.64–0.87)	<0.0001	
**ESBL-producing ** ***E. coli*** **, healthcare-associated**						
I	203/638 (31.8)	II versus I	1.13	(0.90–1.41)	0.280	
II	266/770 (34.5)	III versus I	1.64	(1.33–2.03)	<0.0001	<0.0001
III	393/906 (43.4)	II versus III	0.69	(0.57–0.84)	<0.0001	

ESBL, extended-spectrum β-lactamase.

*P-value is for odds ratio (comparison with reference category I or III).

**P-value is for logistic model.

During the study period, the monthly proportions of ESBL-producing *E. coli* isolates, both community-acquired and healthcare-associated, also increased significantly, whereas in the case of ESBL-producing *K. pneumoniae* isolates, healthcare-associated but not community-acquired isolates increased significantly (Table 2). By contrast, the monthly proportions of carbapenem-resistant *P. aeruginosa* were stable (*P* = 0.648).

### Time-series analyses of carbapenem use and CRAB proportions

**Figure 1:**
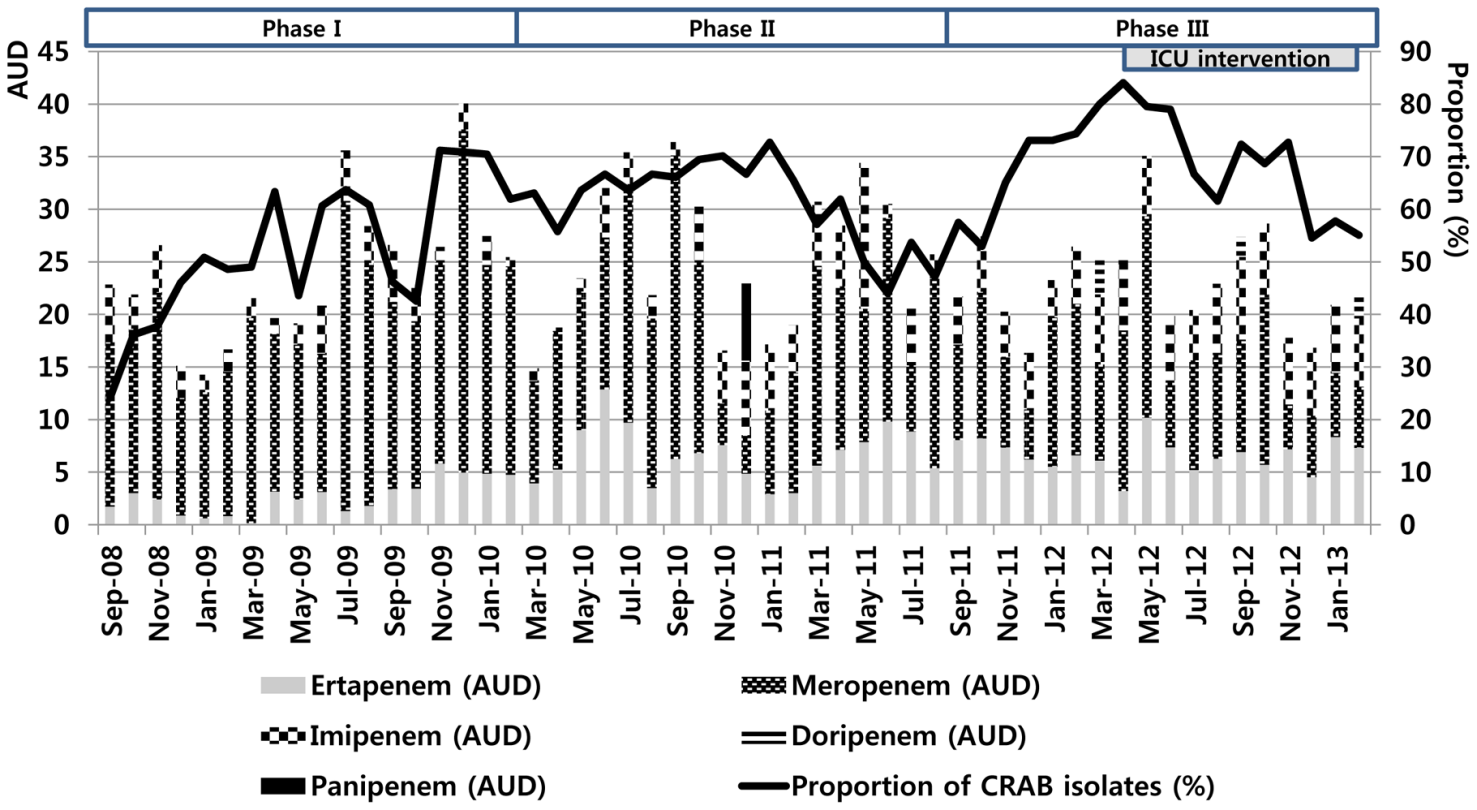
Trends of carbapenem-resistant *Acinetobacter baumannii* isolates (%) and carbapenem use (AUD, defined daily dose per 1,000 patient-days) during the study period.

Linear-regression models that included autoregressive errors were used to assess whether changes in carbapenem consumption affected the susceptibility of *A. baumannii* to carbapenems. Time-series data of the two components examined were available for the entire 54-month period from the hospital infection-control unit (Figure 1). Time-series analyses revealed that an increase in the consumption of either total carbapenems or Group 2 carbapenems during the previous one month was significantly associated with an increase in the proportion of CRAB in the following month. By contrast, an increase in ertapenem consumption during the previous one month was not associated with an increase in the proportion of CRAB in the following month (*P* = 0. 941; Table 3). The resulting models can be represented by the following equations:

**Table 3:** Linear-regression models with autoregressive errors used for examining the effects of carbapenem use on the susceptibility of *Acinetobacter baumannii* to carbapenems.

Variables	Estimate	Standard error	*T* value	*P*-value
**Correlation between carbapenem use and the proportion of CRAB isolates, colonized or infected**				
** Model 1. Total carbapenem use and proportion of CRAB isolates**				
** **Time	0.0028	0.0003	9.69	<0.0001
** **Total carbapenem before one month	0.3442	0.1708	2.02	0.0493
** **AR1	−0.7023	0.0998	−7.04	<0.0001
** **Total R^2^		0.9853		
** Model 2. Group 1 carbapenem use, Group 2 carbapenem use, and proportion of CRAB isolates**				
** **Time	0.0028	0.0003	9.91	<0.0001
** **Ertapenem before one month	−0.0177	0.2358	−0.07	0.9406
** **Group 2 carbapenem before one month	0.3956	0.1777	2.23	0.0306
** **AR1	−0.7295	0.0966	−7.55	<0.0001
** **Total R^2^		0.9852		
**Correlation between carbapenem use and the proportion of CRAB isolates, infected**				
** Model 1. Total carbapenem use and proportion of CRAB isolates**				
** **Time	0.0035	0.0004	9.15	<0.0001
** **Total carbapenem before two months	−0.6113	0.2552	−2.4	0.0203
** **AR1	−0.5480	0.1195	−4.59	<0.0001
** **Total R^2^		0.9545		
** Model 2. Group 1 carbapenem use, Group 2 carbapenem use, and proportion of CRAB isolates**				
** **Time	0.0035	0.0004	9.8	<0.0001
** **Ertapenem before two months	−0.5326	0.3599	−1.48	0.1451
** **Group 2 carbapenem before two months	−0.6618	0.262	−2.53	0.0148
** **AR1	−0.5746	0.1191	−4.82	<0.0001
** **Total R^2^		0.8022		
**Correlation between carbapenem use and the proportion of CRAB isolates, colonized**				
** Model 1. Total carbapenem use and proportion of CRAB isolates**				
** **Time	0.0030	0.0003	8.82	<0.0001
** **Total carbapenem before four months	0.3493	0.2003	1.74	0.0871
** **AR1	−0.6933	0.0997	−6.96	<0.0001
** **Total R^2^		0.9809		
** Model 2. Group 1 carbapenem use, Group 2 carbapenem use, and proportion of CRAB isolates**				
** **Time	0.0030	0.0003	9	<0.0001
** **Ertapenem before four months	0.028	0.2906	0.1	0.9237
** **Group 2 carbapenem before four months	0.3755	0.2136	1.76	0.0848
** **AR1	−0.7153	0.0999	−7.16	<0.0001
** **Total R^2^		0.981		

AR 1, first-order autoregressive errors.

Model 1: Proportion of CRAB isolates (t)  = 0.0028 t+0.3442 Total carbapenem use (t−1) +ν (t)

Model 2: Proportion of CRAB isolates (t)  = 0.0028 t−0.0177 Ertapenem use (t−1) +0.3956 Group 2 carbapenem use (t−1) +ν (t)

where ν (t)  = 0.7023(t−1) + e(t) in Model 1 and ν (t)  = 0.7295 ν (t−1) + e(t) in Model 2, and where ν (t) is a first-order autoregressive error and e(t) is a normally distributed random error.

In the case of patients who were infected with *A. baumannii*, the time-series analyses revealed that an increase in the consumption of either total carbapenems or Group 2 carbapenems during the previous two months was significantly associated with an increase in the proportion of CRAB in the corresponding month. By contrast, an increase in ertapenem consumption during the previous two months was not associated with an increase in the proportion of CRAB in the corresponding month in patients who were infected with *A. baumannii* (Table 3). Moreover, in the case of patients who were colonized with *A. baumannii*, the time-series analyses failed to demonstrate a significant association between carbapenem consumption and the susceptibility of *A. baumannii* to carbapenems (Table 3).

## Discussion

In this before-and-after study, we evaluated how effective a program of carbapenem-use stewardship was in containing CRAB infections at a tertiary-care hospital; we conducted this evaluation by performing time-series analyses of the effect of consuming Group 1 and Group 2 carbapenems on the susceptibility of *A. baumannii* to carbapenems during the 54-month study period. After the program of carbapenem-use stewardship was started during the study's Phase II, preferential use of ertapenem led to statistically significant reductions in the consumption of Group 2 carbapenems, but it did not markedly alter variables relevant to clinical outcomes. Although the susceptibility of *A. baumannii* to Group 2 carbapenems was not enhanced overall during the study period, the results of our time-series analyses demonstrated a statistically significant positive correlation between the proportion of CRAB isolates obtained from infected patients and the use of Group 2 carbapenems. Conversely, we observed no association between the proportion of CRAB isolates obtained from infected patients and the increased use of ertapenem.

According to a Korean nationwide annual surveillance report, the isolation rates of CRAB in hospitals were 13% in 2003, 16% in 2005, 22% in 2007, and 51% in 2009 [6]. In this study, the overall prevalence of CRAB was 60.7%±12.3% per month (median, 63.2%; range, 24.1%–84.1%). Because of the high prevalence of CRAB, strategies that are more effective than those currently available for controlling CRAB in hospitals must continue to be developed. Factors that are predictive of CRAB acquisition have been reported to include old age, a deteriorated functional status, ICU stay, recent invasive procedures or surgeries, prolonged hospital stay, immunosuppression, and antibiotic exposure [9]. Of particular interest is that prior exposure to imipenem has been reported as the main risk factor for CRAB acquisition [20]–[22]. In vitro, imipenem might potently induce MDR *A. baumannii* strains [8]. However, few ecological trials have analyzed how exposure to carbapenems affects the proportion of CRAB or determined the precise effects of carbapenem-use stewardship practices. Previously, a positive correlation was demonstrated between the use of anti-pseudomonal carbapenems and the rate of CRAB isolation in the hospital setting [23]–[25]. However, other studies have raised concerns regarding the impact of ertapenem use on the selection of ertapenem-resistant bacteria and also of bacteria that exhibit cross-resistance to Group 2 carbapenems [11], [12].

Our findings demonstrated that the mandatory use of ertapenem could replace the prescription of Group 2 carbapenems in the case of patients with ertapenem-susceptible gram-negative bacilli under an antibiotic-stewardship program, which would subsequently reduce the ecological selection pressure among *A. baumannii* isolates. However, isolation rates of CRAB increased continuously during the implementation of the antibiotic-stewardship program. Similarly, a statistically significant reduction in CRAB rates was not observed previously following the implementation of a comprehensive antibiotic-stewardship program [15], [22], [26]. These findings suggest that the development of antibiotic resistance is multifactorial. Furthermore, infection-control practices, prolonged hospital or ICU stays, exposure to invasive procedures, comorbidities of patients, and advanced age might also be confounders in this analysis. Specifically, cross-transmission between patients—through transient colonization on healthcare workers' hands and environmental contamination sources—was reported to be a major contributing factor for the rise in CRAB infections [27]. In Phase III of our study (April to December 2012), environmental surveillance cultures were prepared and a medical ICU was subjected to intensive environmental cleaning, and this is likely associated with a decrease in proportion of CRAB isolates from 84.1% in April 2012 to 54.5% in December 2012. Furthermore, the increasing prevalence of ESBL-producing *E. coli* and ESBL-producing *K. pneumoniae* in our hospital might have affected the association of carbapenem use with the proportion of CRAB isolates [10].

In the study, autoregressive-error models were used for analyzing the relationship between the consumption of Group 1 and Group 2 carbapenems and the susceptibility of *A. baumannii* to carbapenems before and after the introduction of our carbapenem-use stewardship program. This study, like previous studies, demonstrated an association between the increased use of Group 2 carbapenems and increased rates of CRAB isolates; by contrast, no statistically significant association was observed between the consumption of ertapenem and the rates of CRAB isolates [16], [24], [25]. These findings suggest that ertapenem use did not affect the cross-resistance of nonfermentative gram-negative bacilli to Group 2 carbapenems, and this had a positive impact on the hospital ecology by suppressing the selection pressure exerted by Group 2 carbapenems [14], [26].

This study had a few potential limitations. First, the experimental design of this before-and-after study is prone to bias mainly because of the failure to control for potential confounding variables that affect antimicrobial resistance in the hospital setting. Second, the use of the VITEK II system failed to distinguish *A. baumannii* from the *A. baumannii-calcoaceticus* complex at the level of genomospecies. Thus, the inability to differentiate the genomospecies of *A. baumannii* isolates might result in an overestimation of the prevalence of *A. baumannii*.

In conclusion, the preferential use of ertapenem in the case of infections that require carbapenem treatment resulted in reduced use of Group 2 carbapenems, which positively affected the susceptibility of *A. baumannii* to carbapenems. A carbapenem-use stewardship program that includes the replacement of Group 2 carbapenems with ertapenem should be considered as one of the multifaceted infection-control strategies used for reducing or containing the spread of CRAB in hospitals.
